# Isolated Ruptured Pulmonary Hydatid Cyst Mimicking Community-Acquired Pneumonia

**DOI:** 10.7759/cureus.95612

**Published:** 2025-10-28

**Authors:** Khawla Y Alawadhi, Sara A Alkhoori, Shaikha A Alshamsi, Sarah Baroud, Rasha Awawdeh, Rami Almadi

**Affiliations:** 1 Internal Medicine, Mohammed Bin Rashid University of Medicine and Health Sciences (MBRU), Dubai, ARE; 2 Internal Medicine, Mediclinic Parkview Hospital, Dubai, ARE; 3 Pulmonary Medicine, Mediclinic Parkview Hospital, Dubai, ARE

**Keywords:** echinococcus granulosus, hydatid disease, pneumonia, ruptured pulmonary hydatid cyst, water-lily sign

## Abstract

Hydatid disease, most commonly caused by *Echinococcus granulosus*, usually affects the liver, and isolated pulmonary involvement is rare. Ruptured lung cysts can mimic pneumonia, often delaying diagnosis. Although surgery is the standard treatment, medical therapy alone has occasionally been successful. We report a 42-year-old healthy Lebanese man who presented with fever and elevated inflammatory markers suggestive of pneumonia that did not respond to broad-spectrum antibiotics. Imaging revealed right lower lobe consolidation accompanied by bilateral patchy ground-glass opacities. Additionally, there was a cavitary lesion with detached membranes in the right lower lobe, consistent with a ruptured pulmonary hydatid cyst. No liver involvement was noted. He was treated conservatively with albendazole and antibiotics, leading to rapid clinical and biochemical improvement. This case highlights the need to consider hydatid disease in endemic regions for non-resolving pneumonia and demonstrates the potential role of medical therapy alone in select patients.

## Introduction

Hydatid disease, or cystic echinococcosis, is a parasitic infection caused by *Echinococcus granulosus*, with humans serving as accidental intermediate hosts. Infection occurs through ingestion of eggs via contaminated food, water, or direct contact with dogs [1]. The liver is the most commonly affected organ, accounting for 70% of cases, while pulmonary involvement occurs in 20% [2]. Clinical manifestations vary depending on the organ involved; pulmonary cysts may be asymptomatic and detected incidentally or cause cough, chest pain, dyspnea, or hemoptysis [1]. Rupture can potentially mimic pneumonia [3] or lead to secondary bacterial infection [4], delaying appropriate management. Isolated pulmonary hydatid cysts without hepatic disease are uncommon and can present diagnostic challenges [5]. Although surgical removal is the standard treatment, medical therapy with albendazole can be successful in select cases [1,4,5]. We present the case of a 42-year-old healthy Lebanese man with a ruptured solitary pulmonary hydatid cyst initially masquerading as community-acquired pneumonia, who improved with medical therapy alone. This case underscores the importance of considering hydatid disease in endemic regions when pneumonia is non-resolving and highlights the potential role of medical therapy in selected patients.

## Case presentation

A 42-year-old previously healthy Lebanese male presented to the emergency department with a two-day history of fever, chills, and malaise, initially without respiratory symptoms. He attributed the onset of his symptoms to recent exposure to cold air conditioning at an event. The review of systems was otherwise unremarkable, with no cardiovascular, gastrointestinal, neurological, genitourinary, or dermatological complaints. He denied recent sick contacts or known allergies and reported no history of smoking.

His medical history is notable only for a COVID-19 infection in April 2020 (five years prior to this presentation), during which chest X-ray and computed tomography (CT) chest scans showed no evidence of pulmonary hydatid cysts. He has no significant surgical history. He currently resides in Dubai, United Arab Emirates, but has returned annually to Lebanon for one-month visits over the past five years, with the most recent trip one month prior to presentation. Although he lives in Beirut, he occasionally visits relatives in rural areas where dogs and livestock are present. He denied direct contact with animals and consistently consumed bottled water during his visits; however, he regularly ate raw vegetables such as parsley, commonly used in tabbouleh salad.

On examination, he was alert, oriented, and in no acute distress. His temperature was 39.4°C, heart rate 120 bpm (regular), blood pressure 121/75 mmHg, respiratory rate 16/min, and oxygen saturation 98% on room air. His BMI was 29.8 kg/m². He appeared mildly dehydrated. Chest auscultation revealed decreased air entry at the right lower lung base with crepitations but no additional sounds. The remainder of the physical examination was unremarkable.

Initial laboratory studies revealed leukocytosis with neutrophil predominance (white blood cell count: 18.2 × 10³/μL; neutrophils: 84.1%) without eosinophilia, and a markedly elevated C-reactive protein (307 mg/L). Kidney function, liver function, and serum electrolytes were within normal limits (Table 1). A chest X-ray demonstrated a homogeneous right lower lobe opacity with air bronchograms and blunting of the right costophrenic angle, suggestive of a pleural effusion.

**Table 1:** Laboratory results on admission

Lab marker	Finding	Reference range
White blood cell count	18.5 × 10³/μL	4.0-11.0 × 10³/μL
Neutrophils	84.15%	43.5-73.5%
Eosinophils	0.49%	0.8-8.10%
Red blood cell count	4.65 × 10⁶/μL	4.50-5.90 × 10⁶/μL
Hemoglobin	14.3 g/dL	13.0-17.5 g/dL
Platelet count	186 × 10³/μL	150-450 × 10³/μL
C-reactive protein	307 mg/L	0-5 mg/L
Creatinine	102 μmol/L	62.0-106.0 μmol/L
Estimated glomerular filtration rate	81 mL/min/1.73 m²	>60 mL/min/1.73 m²

Based on the clinical presentation and initial findings, the patient was admitted with a provisional diagnosis of right lower lobe community-acquired pneumonia and was started on empiric intravenous ceftriaxone (2 g once daily) and azithromycin (500 mg once daily).

During the first 24 hours of admission, the patient continued to experience high-grade fever spikes and developed a productive cough with yellowish sputum. Examination revealed increased work of breathing, persistent right lower lobe crackles, and stony dullness to percussion. Inflammatory markers remained elevated, with C-reactive protein (CRP) rising from 307 mg/L on admission to 418 mg/L. In view of his clinical deterioration, empiric antibiotics were escalated from ceftriaxone (2 g once daily) to intravenous piperacillin-tazobactam (4.5 g every 6 hours) in combination with azithromycin (500 mg once daily). A comprehensive microbiological workup, including blood, urine, and sputum cultures, a respiratory viral panel, *Legionella* urinary antigen, *Mycoplasma* testing, and a pneumonia molecular panel, was sent.

Over the next 48 hours, fever persisted and CRP continued to rise, while the white blood cell count fluctuated between 13-15 × 10⁹/L. Notably, the eosinophil percentage had progressively increased from 0.49% on admission to 3.40%. All microbiological studies returned negative. In light of these findings, intravenous linezolid (600 mg every 12 hours) was added. Due to concern for a parapneumonic effusion, a chest ultrasound was performed, which revealed only a small right-sided pleural effusion (~35 mL), not amenable to drainage.

**Figure 1:**
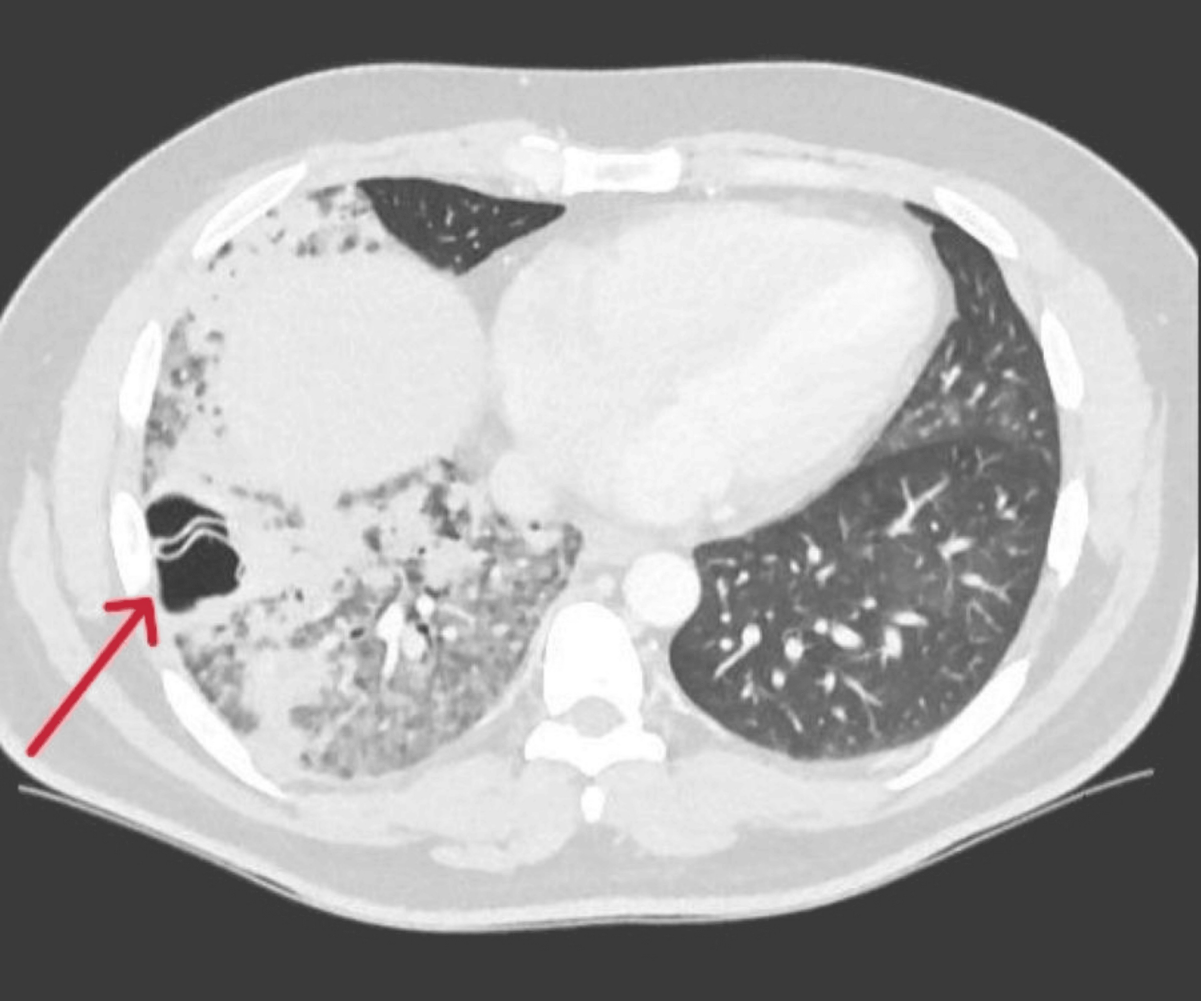
CT scan of the chest (axial view) demonstrating a cavitary lesion with a detached internal membrane in the lateral basal segment of the right lower lobe with surrounding consolidation. The arrow indicates a large cavity with irregular internal membranes floating inside the right lung. This appearance is sometimes called the “water lily sign," which is classic for a ruptured pulmonary hydatid cyst.

By 72 hours after admission, CRP had peaked at 570 mg/L. A contrast-enhanced CT chest was performed, which demonstrated right lower lobe consolidation with patchy ground-glass opacities in the right upper lobe, lingula, and basal segments of the left lower lobe. A cavitary lesion that measured 4 x 2.9 cm with a detached internal membrane was identified in the lateral basal segment of the right lower lobe, consistent with a ruptured pulmonary hydatid cyst (Figure 1). Multiple enlarged mediastinal lymph nodes were seen, located in the right paratracheal, subcarinal, precarinal, and aortopulmonary regions, as well as in the right hilar region. No significant pleural effusion was present. Other thoracic and upper abdominal structures were unremarkable. An abdominal and pelvic ultrasound showed no hepatic or intra-abdominal cysts.

A multidisciplinary discussion with the pulmonology and thoracic surgery teams was held, and a decision was made to initiate albendazole (400 mg every 12 hours) alongside continued antibiotics, given the classical CT findings and the patient’s clinical stability. Surgery was deferred, with the plan to reconsider operative intervention if follow-up imaging demonstrates persistent disease or if symptoms fail to improve with medical therapy.

Within 48 hours of albendazole therapy, the patient’s fever resolved, C-reactive protein (CRP) decreased from 570 mg/L to 140 mg/L, and his symptoms improved significantly. The eosinophil count, which had shown a notable increase during hospitalization, continued to rise until discharge. Despite this, he remained hemodynamically stable throughout his admission and was discharged on hospital day six in stable condition, afebrile, and maintaining oxygen saturation of 95% on room air. On examination, residual crepitations persisted at the right lung base. CRP had declined further to 87.6 mg/L (Table 2).

**Table 2:** Lab marker trend during hospitalization

Lab markers	On Admission	Inpatient Day 1	Inpatient Day 2	Inpatient Day 3	Inpatient Day 4	Inpatient Day 5	Inpatient Day 6 (Discharge day)	Reference range
White Blood Cell Count	18.2 × 10³/μL	16.6 × 10³/μL	13.9 × 10³/μL	14.6 × 10³/μL	15.0 × 10³/μL	10.5 × 10³/μL	11.9 × 10³/μL	4.0-11.0 × 10³/μL
Eosinophil Count	0.49 %	2.67 %	3.40 %	0.60 %	5.99%	10.06%	10.23 %	0.8-8.10%
C-Reactive Protein	307 mg/L	418 mg/L	473 mg/L	570 mg/L	333 mg/L	140 mg/L	87.6 mg/L	0-5 mg/L

He was discharged on albendazole (400 mg twice daily), alongside linezolid (600 mg twice daily) and levofloxacin (750 mg once daily). The antibiotic course was set for 10 days. At follow-up in the pulmonology clinic one week later, he was clinically well and stable. A chest X-ray showed improvement in the right lower lobe pneumonia, while the cavity and surrounding infiltration remained stable. He was advised to continue albendazole for an additional 3-4 weeks, with the total duration to be determined based on follow-up CT imaging and clinical response during reassessment in the thoracic surgery clinic.

Notably, serologic testing for *Echinococcus granulosus* antibodies, sent during admission, returned positive after discharge, confirming the radiological diagnosis.

## Discussion

A hydatid pulmonary cyst is a cystic lesion in the lung parenchyma caused by infection with the larval stage of the tapeworm *Echinococcus granulosus*, a zoonotic pathogen. Humans are accidental intermediate hosts, acquiring the infection through ingestion of eggs via contaminated food, water, or direct contact with dogs [1]. Endemic areas include the Middle East, the Mediterranean Basin, South America, Central Asia, and parts of Africa and Australia [1-2].

Diagnosis relies primarily on imaging, which reveals characteristic cystic or infiltrative lesions in the liver, lungs, and other organs [1]. On chest radiography, an uncomplicated hydatid cyst appears as a well-defined, homogeneous opacity sharply demarcated from the surrounding lung parenchyma. The cyst size can range from 1 to 20 cm, with peripheral cysts often larger than centrally located ones. Differential diagnoses include fluid-filled cysts, benign tumors, carcinoma, metastases, and inflammatory masses. Adjacent atelectatic or reactive changes may obscure cyst margins, mimicking pneumonia or carcinoma. Calcification within pulmonary cysts is rare. CT thorax plays an important role in complicated cases. When a cyst ruptures into a bronchus, air enters between the pericyst and endocyst, leading to characteristic radiologic appearances. The “crescent sign” (radiolucent rim) is an early feature but is not specific, as it can also occur in mycetoma, blood clots, carcinoma, and Rasmussen’s aneurysm. Progressive air entry produces the “cumbo” or “double arch sign,” while collapse of the endocyst gives rise to membranes floating in the remaining fluid, known as the “water lily” or “camolette sign.” With secondary infection, air-fluid levels may develop, closely mimicking a lung abscess. The role of ultrasound is limited except when lesions are close to the thoracic wall, and it is also useful for detecting concomitant liver involvement [3,4]. Serology can further support the diagnosis by detecting specific *Echinococcus* antigens, while in seronegative or complex cases, biopsy (via bronchoscopy), polymerase chain reaction (PCR), and fluorodeoxyglucose positron emission tomography (FDG-PET) may be used to confirm infection and assess lesion activity [1,4].

A ruptured pulmonary hydatid cyst, particularly when it ruptures into the bronchus, can initially cause allergic pneumonitis, which is typically reversible within 10 days. If infection occurs, this may progress to secondary bacterial pneumonia after 10 to 14 days. Peripheral blood eosinophilia is present in less than 25% of infected individuals and tends to be more common with cyst rupture. Leukocytosis and increased erythrocyte sedimentation rate are also observed, but these findings are nonspecific and can be elevated in a variety of conditions [4].

Isolated pulmonary hydatid disease, without concomitant hepatic involvement, is uncommon, with few cases reported in the literature [5-7]. Surgical excision remains the standard treatment, with procedures such as cystotomy with capitonnage or pericystectomy aimed at removing the cyst while preserving lung parenchyma; lobectomy is reserved for extensive disease [4,8]. Studies have shown that surgery offers low morbidity and recurrence rates, making it the preferred approach in most cases. For example, a retrospective cohort study at Lady Reading Hospital in Peshawar found that lung-sparing procedures like cystotomy with capitonnage were associated with superior outcomes, including shorter hospital stays, lower morbidity, and no mortality, highlighting the benefits of conservative surgical management, especially in endemic and resource-limited settings [9]. Similarly, a single-centre analysis of 872 pulmonary hydatid cyst cases reported cystotomy with capitonnage in 782 patients, with low percentages of postoperative complications including atelectasis (5.6%), prolonged air leak (1.0%), empyema (0.7%), wound infection (0.4%), and bleeding (0.2%), a recurrence rate of only 0.5%, and postoperative mortality of 0.1% [10].

In contrast, medical treatment as sole therapy remains debated. Benzimidazole anthelmintics, such as albendazole, are primarily used pre- and post-operatively to reduce recurrence but may be employed alone in selected cases, particularly when surgery carries increased risk due to comorbidities, anatomical challenges, or early rupture risk [4,8]. Several reports in the literature highlight albendazole as a viable alternative to surgery. Talwar et al. (1990) demonstrated albendazole’s effectiveness as a primary treatment in pulmonary hydatid disease, especially in nonsurgical candidates, with good clinical and radiological improvement [11]. Galanakis et al. (1997) reported successful treatment of complicated pulmonary hydatid cysts in children using albendazole alone, supporting its role as a non-surgical option in selected pediatric cases [12]. Sheikhy et al. (2015) described variable and unpredictable clinical outcomes of ruptured pulmonary hydatid cysts managed medically, underscoring the need for individualized treatment plans and close follow-up [13]. A study by Doğru et al. (2005) in pediatric pulmonary hydatid disease showed that medical treatment with albendazole or mebendazole is safe and effective, particularly for uncomplicated cysts smaller than 5 cm. Among 82 children treated, 34.1% were cured, 34.1% improved, and 31.8% failed treatment; analysis of 102 cysts showed similar results. While cure and failure rates were comparable between albendazole and mebendazole, albendazole was associated with significantly more cases of improvement [14]. A case report by Hejazi et al. (2016) described successful medical management of multiple pulmonary hydatid cysts using albendazole alone, with four cycles over five months leading to significant clinical, radiological, and serological improvement and no relapse after 21 months of follow-up [15].

The PAIR (puncture, aspiration, injection, re-aspiration) technique is a minimally invasive, image-guided procedure used selectively for small, uncomplicated pulmonary hydatid cysts. It involves aspirating cyst fluid and injecting a scolicidal agent (e.g., hypertonic saline or ethanol) to eliminate the parasite, often combined with albendazole to reduce recurrence. However, PAIR is contraindicated in cysts adjacent to major blood vessels, with active biliary communication, large or infected cysts, multilocular or heavily calcified cysts, and in certain patients, such as pregnant women or those with significant comorbidities, where surgery or medical therapy remains preferable [16].

Overall, while surgery remains the gold standard due to its low morbidity and recurrence, medical therapy with benzimidazoles such as albendazole represents a valuable alternative for carefully selected patients, particularly those at high surgical risk or with multiple inoperable cysts. Close clinical and radiological follow-up is essential to monitor for potential relapse.

This case is notable for several reasons. The patient initially presented with minimal respiratory symptoms and fever, leading to a provisional diagnosis of community-acquired pneumonia, with no evidence of a hydatid cyst on the initial chest X-ray. However, despite broad-spectrum antibiotics, inflammatory markers remained persistently elevated, and clinical deterioration prompted further evaluation. A CT scan revealed right lower lobe consolidation alongside the characteristic “water lily sign,” indicating a ruptured pulmonary hydatid cyst, an uncommon finding given the absence of hepatic involvement. A secondary bacterial infection likely followed the rupture, which can be explained by the worsening clinical picture and subsequent rise in eosinophils. Although surgery is typically the preferred treatment, the patient’s clinical stability, absence of severe complications, and clinical improvement as reflected by a progressive decline in CRP that paralleled stabilizing imaging findings following albendazole therapy, supported a conservative medical approach. This outcome highlights the importance of considering hydatid disease in endemic regions for cases of non-resolving pneumonia and suggests that medical therapy alone can be effective in carefully selected patients. Repeat imaging and follow-up are planned to document further resolution and guide ongoing management.

## Conclusions

This case emphasizes the importance of considering pulmonary hydatid disease, even in its isolated form, in the differential diagnosis of non-resolving pneumonia in endemic regions, and highlights that while surgery remains the mainstay of treatment, albendazole monotherapy may be an effective alternative in carefully selected, clinically stable patients.
